# Investigation of the molecular mechanism of Xiaoluo Wan in thyroid-associated ophthalmopathy: network analysis and in vivo study

**DOI:** 10.1186/s41065-025-00499-0

**Published:** 2025-07-22

**Authors:** Wenchao Gu, Shuo Tian, Lu Fu, Lina Wang, Liangkun Zhang, Furong Wang

**Affiliations:** 1 College of Traditional Chinese Medicine, Shandong University of Traditional Chinese Medicine, Jinan, 250355 China; 2 Medical College, Shandong University of Traditional Chinese Medicine, Jinan, 250355 China; 3 Department of Traditional Chinese Medicine, Jinan Central Hospital Affiliated to Shandong First Medical University, Jinan, 250013 China

**Keywords:** Xiaoluo wan, Thyroid-associated ophthalmopathy, Network analysis, Oxidative stress, HIF1 signaling pathway

## Abstract

**Background:**

Thyroid-associated ophthalmopathy (TAO) is a common complication of hyperthyroidism that can significantly impair quality of life. This study investigated the effects and mechanisms of Xiaoluo Wan (XLW), a traditional Chinese herbal prescription, in treating TAO.

**Methods:**

The protective effects of XLW on the extraocular muscles were first examined in hyperthyroid rats. Network analysis strategies were applied to predict potential targets and therapeutic mechanisms associated with XLW. The expression of key genes and proteins was subsequently validated and analyzed in rats with hyperthyroidism.

**Results:**

XLW alleviated the pathological changes in the extraocular muscles caused by hyperthyroidism. The network analysis identified 66 effective targets. The core targets of XLW against TAO included AKT1, PTGS2, BCL2, IL10, IL1b, CCL2, IFNG, IL6, MMP9, TGFB1, HIF1α, and TP53. Enrichment analysis suggested that the amelioration mechanisms of XLW may be linked to the HIF1 signaling pathway. In hyperthyroid rats, XLW reduced oxidative stress (OS) in extraocular muscle and inhibited the expression of HIF-1ɑ. Additionally, XLW exerted regulatory actions on the expression of various proteins closely linked to HIF-1α and OS.

**Conclusions:**

XLW reduces injuries to extraocular muscles in hyperthyroidism, possibly by inhibiting OS via HIF1 signaling. This may provide novel insights into the pharmacological mechanism of XLW in treating TAO.

**Supplementary Information:**

The online version contains supplementary material available at 10.1186/s41065-025-00499-0.

## Introduction

Thyroid-associated ophthalmopathy (TAO) is a complex inflammatory disorder of the eye that affects the orbit and adjacent facial tissues [1]. Key clinical features of TAO include disfigurement, redness, eyelid pain, swelling, a gritty feeling in the eyes, double vision, and occasionally blindness. These symptoms can significantly affect physical and emotional functions, reduce quality of life, and diminish work capacity [2–4]. The annual incidence rate of TAO is 16 women and 3 men per 100,000 people [5].

The pathogenesis of TAO remains unclear. An imbalance between production and removal of reactive oxygen species (ROS) leads to oxidative stress (OS), causing ROS-mediated damage to cells and their processess [6]. Recent in vivo and in vitro studies have demonstrated a strong correlation between OS and TAO [7–9]. ROS stimulates the proliferation of orbital fibroblasts and mucopolysaccharides. Moreover, it upregulates the expression of inflammatory factors and other mediators, including intercellular adhesion molecule-1 and human leukocyte antigen [10]. All of these factors can contribute to TAO progression.

There are various treatment options available based on the severity of the disease. For patients with mild disease, local treatments are appropriate. Conversely, for those with moderate to severe disease activity, intravenous glucocorticoids or immunosuppressants are recommended. However, these medications are associated with varying degrees of side effects [11]. Currently, there are no clinically proven drugs for TAO prevention and treatment that have been validated in randomized, placebo-controlled multi-center trials.

Traditional Chinese medicine (TCM) has emerged as a novel therapeutic paradigm for managing TAO. It is essential to identify safe and effective agents with multi-component and multi-target characteristics from herbal medicine resources to help reduce the recurrence of diseases. Xiaoluo Wan (XLW), a TCM decoction, is made up of four crude herbs: *Scrophularia ningpoensis* Hemsl. (Xuanshen, XS), *Prunella vulgaris* (Xiakucao, XKC), *Fritillaria thunbergii* (Zhebeimu, ZBM), and Oysters (Muli, ML) (Table 1). According to TCM theories, XLW exerts effects such as softening hardness to dissipate stagnation (Ruanjian Sanjie), nourishing yin to lessen fire (Ziyin Jianghuo). Numerous studies have indicated that XLW and its components can improve the clinical symptoms of thyroid diseases [12–14]. However, these studies failed to identify the primary targets and pathways of XLW against TAO. Given the potential significance of XLW in managing TAO, it is essential to further investigate its mechanisms and provide evidence for its clinical application.

**Table 1:** The ingredients of XLW

China name	Abbreviations	Latin binomial nomenclature	Part of use	Proportion
Xuanshen	XS	*Scrophulariae radix*	Root	1
Xiakucao	XKC	*Prunellae spica*	Bulb	2
Zhebeimu	ZBM	*Fritillariae thunbergii bulbus*	Ear	1
Muli	ML	*Ostreae concha*	Shell	2

**Fig. 1:**
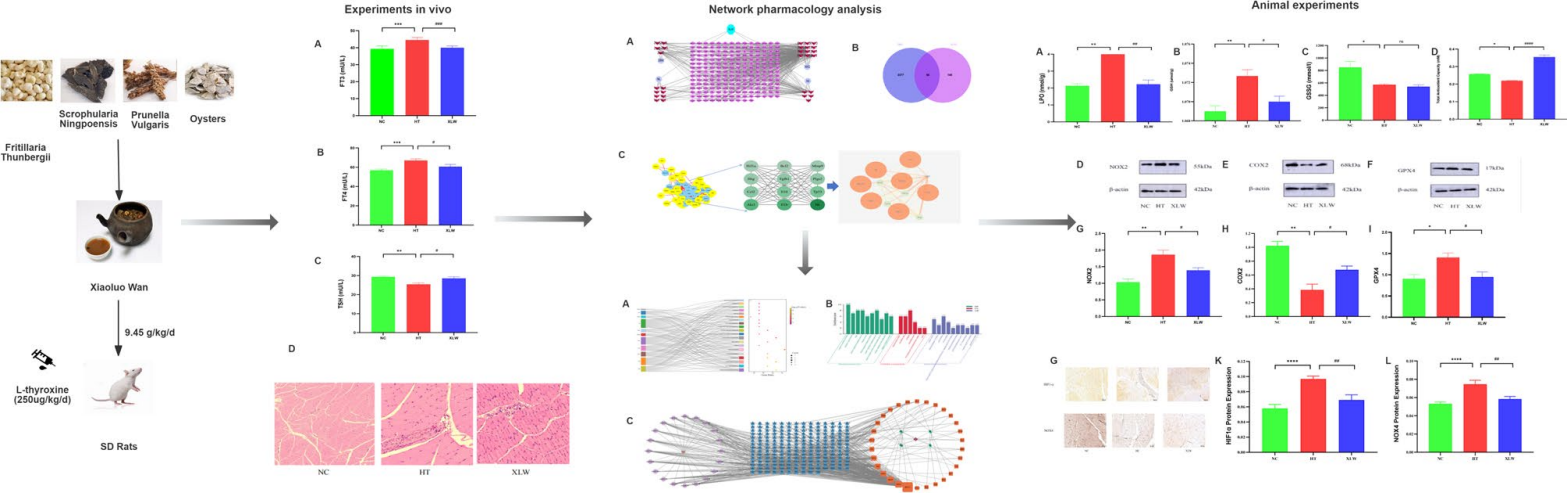
Schematic illustration of the animal experimental design

Consequently, this research aimed to initially assess the beneficial influence of XLW on extraocular muscles in hyperthyroid rats. Subsequently, the network analysis was conducted to forecast the potential molecular targets and bio-active ingredients. Additionally, the primary mechanisms were validated in hyperthyroid rats. These findings may provide a basis for the clinical application of XLW in TAO therapy. The study schedule is depicted in Fig. 1.

## Materials and methods

### Materials

Herbs ML (lot: 22,042,101), XS (lot: 20,220,303), and XKC (lot: 20,220,412) were procured from Shandong Shunshengtang Chinese Medicine Pieces Co., Ltd. The ZBM (lot: 22,042,101) was obtained from Shandong Baiweitang Chinese Medicine Pieces Co., Ltd. Additionally, free triiodothyronine kit (FT3; JM-10758R2), free thyroxine kit (FT4; JM-01990R2), and thyroid-stimulating hormone kit (TSH; JM-10912R2) were provided by Jingmei Biotechnology, Jiangsu, China. The total antioxidant capacity (T-AOC) kit (S0119) was obtained from Beyotime Biological Co., Ltd (Shanghai, China). The glutathione (GSH) kit (R22075) and glutathione disulfide (GSSG) kit (R22228) were procured from Yuanye Bio-Technology Co., Ltd (Shanghai, China). The lipid peroxidation (LPO) kit (BC5240) was provided by Solarbio Science&Technology Co., Ltd. (Beijing, China). The acquisitions of anti-rat antibodies are as follows: NOX2 (sc-130543, Santa Cruz Biotechnology, Santa Cruz, USA), GPX4 (T56959F, Abmart, Shanghai, China), PTGS2 (66351-1-Ig, Proteintech, Wuhan, China), HIF1α (PB9253, BOSTER, Wuhan, China), NOX4 (#DF6924, Affinity Biosciences, Jiangsu, China), and β-actin (20536-1-AP, Proteintech, Wuhan, China). HRP-labeled Goat anti-Mouse IgG (S0001) and HRP-labeled Goat anti-Rabbit IgG (BS12478) were obtained from Affinity Biosciences and Bioworld Technology (Minneapolis, MN, USA), respectively.

### Animals

A total of 22 SPF male SD rats weighing 180–220 g were procured from Beijing Vital River Laboratory Animal Technology Co., Ltd. (License number: SCXK [Beijing] 2021-00060). All animal experiments were conducted in full compliance with the International Ethical Guidelines, with approval from the Committee for Animal Ethics of Shandong University of TCM (Approval Date 09-10-2021, Approval No. SDUTCM20211009001).

### Preparation of XLW

The herbs in XLW were prepared in a 2:1:1:2 ratio. All herbs were soaked in ultra-pure water for 2 h. ML was initially pre-decocted in ultra-pure water for 30 min, followed by the addition of XS, ZBM, and XKC. The mixture was then decocted for an additional hour. The solution was filtered, and an equal volume of ultra-pure water was added to the herbs for another hour of decoction. The two decoctions were combined into a single solution. Finally, the extract was concentrated and stored at 4 ℃.

### Animal treatments

All rats were allowed unlimited access to food and water. Following a seven-day acclimation period, the animals were randomly assigned to three groups: The normal group (NC) (*n* = 6), the hyperthyroidism group (HT) (*n* = 8), and the XLW group (*n* = 8). Rats in the HT and XLW groups received intraperitoneal injections of L-thyroxine (250 µg/kg/d). Three days later, the rats in NC and HT groups were administered saline, while rats in the XLW group were given XLW by gastric gavage once daily (9.45 g/kg/d) for five consecutive weeks.

### Histopathologic examinations

All rat extraocular muscle tissue specimens were fixed with 4% paraformaldehyde to make paraffin-embedded blocks. After baking, the slices were affixed to a dyeing rack. Hematoxylin and eosin (H&E) staining was employed to examine the arrangement of the extraocular muscles. The changes were observed using an advanced upright fluorescence microscope.

### Thyroid function test

To determine whether XLW affects thyroid function, the concentrations of thyroid hormones in the peripheral blood, including FT3, TSH, and FT4, were measured using enzyme-linked immunosorbent assay (ELISA). The procedure was carried out according to the kit instructions.

### Network pharmacology-based analysis

#### Screening of active ingredients of XLW

The active components of XS, XKC, and ZBM in XLW were searched through the TCMSP database (https://old.tcmsp-e.com/tcmsp.php), while ML was searched using the TCMID database (https://ngdc.cncb.ac.cn/databasecommons/database/id/437). Only ingredients with an oral bioavailability (OB) of 30% or higher and a drug-likeness (DL) score of 0.18 or above were included; others were excluded. Relevant databases were consulted to identify the key herbal-related active ingredients screened in the TCMSP database.

### Acquisition of candidate therapeutic targets of XLW

The active ingredients were submitted to the PubChem database (https://pubchem.ncbi.nlm.nih.gov/) [15] to obtain their structures and canonical SMILES. They were then uploaded to the SwissTargets database (http://www.swisstargetprediction.ch/) [16] to identify the targets, which were calibrated by the UniProt database (https://www.uniprot.org/) [17].

### Collection of related targets for TAO

TAO-related targets were identified using the GeneCards database (https://www.genecards.org/) [18], the OMIM database (https://www.omim.org/) [19], the DisGeNET database (https://www.disgenet.org/home/) [20], and the Drugbank database (https://www.drugbank.ca/) [21]. The acquired data were calibrated with the UniProt database (https://www.uniprot.org/) [22].

### Construction of the PPI network

To clarify the interaction between TAO-related targets and herb targets, we used Venny (version 2.1) (http://bioinfogp.cnb.csic.es/tools/venny/index.html) software to identify overlapping targets by entering the disease and herb targets. The potential targets were uploaded to the STRING database (https://string-db.org/cgi/input.pl) [23] to construct a PPI network. We applied the following conditions: “Rattus norvegicus” and “high confidence (0.700)” to gather potential target information. Finally, target information was input into the Centisape (version 2.2) to identify the kernel targets based on the scores of Betweenness, Closeness, and Degree.

### Functional enrichment analysis

The DAVID database (https://david.ncifcrf.gov/) is a useful web-based tool for annotating gene functions. In this study, we conducted gene ontology (GO) function enrichment and Kyoto encyclopedia of genes and genomes (KEGG) pathway enrichment analysis. The results were visualized using online bioinformatics tools to further investigate potential therapeutic targets, their biological functions, and key pathways. GO analysis describes the function of gene products. Ultimately, we screened the top 10 items of GO analysis and the top 20 of KEGG (*p* < 0.05).

### Experimental validation of potential targets

Preparation of XLW, animal induction, the TAO model, and drug treatments were conducted following the experimental protocol outlined above. After treatment, the rats underwent a 24-h fasting before receiving an intraperitoneal injection of 20% urethane at a dosage of 1.2 g/kg for anesthesia. Blood from the abdominal aorta was collected and spun for 15 min to acquire serum for thyroid function analysis. The extraocular muscles were excised, fixed in 4% paraformaldehyde, and embedded in paraffin for histopathological examination. Another portion of the extraocular muscle was immediately frozen in liquid nitrogen, stored at − 80 ℃, and utilized for Western blotting (WB) analysis.

### ELISA

We employed ELISA to measure T-AOC, GSH, GSSG, and membrane LPO levels in tissue. The extraocular muscle samples were washed twice with prechilled PBS, then homogenized while kept cool and centrifuged at 4 ℃. The supernatant was carefully removed afterwards. The ELISA kit was thawed to room temperature with gentle mixing before processing the samples. Data analysis was conducted following the manufacturer’s guidelines.

### WB

Protein expressions of NOX2, GPX4, and PTGS2 were measured in rat extraocular muscle by WB. Protein from extraocular muscle tissue was extracted using PIPA lysis solution combined with 1 M PMSF, 10% cocktail, and phosphatase-protease inhibitor. The concentration was determined with a BCA protein assay kit, followed by centrifugation and denaturation. Proteins were separated via SDS-PAGE and transferred to PVDF membranes. Membranes were blocked in 5% BSA for 1 h at room temperature, followed by incubation with primary antibodies overnight at 4 ℃ and secondary antibodies for 1 h at room temperature. Finally, we developed the color using enhanced chemiluminescent reagent luminescence and calculated the relative protein levels using ImageJ software. Primary antibodies used included the following: Anti-NOX2 (1:1000), Anti-GPX4 (1:2000), Anti-PTGS2 (1:12000), and β-actin (1:15000). Secondary antibodies utilized were HRP-labeled Goat anti-Mouse IgG (1:10,000) and HRP-labeled Goat anti-Rabbit IgG (1:100,000).

### Immunohistochemical staining

The impact of XLW on HIF1α and NOX4 expression in the extraocular muscle tissue of rats treated with L-thyroxine was analyzed using immunohistochemistry. Antigen repair of extraocular muscle tissue sections was performed using a microwave oven. The tissues were roasted, made transparent, dehydrated, washed with PBS after cooling, and then sealed. Nonspecific binding sites were blocked by incubation with serum from the same species as the secondary antibody. A circle was drawn around each tissue using an immunohistochemistry pen, followed by the application of diluted serum, which was incubated at 37 ℃. Next, we added 20 µL of primary antibodies HIF1α (1:100) and NOX4 (1:100) and incubated overnight at 4 ℃ in a refrigerator. After three washes with PBS, the sections underwent a second incubation with secondary antibody at room temperature for 30 min. For color development, DAB substrate was applied, and the reaction was terminated by washing the sections under running water. The slices underwent hematoxylin re-staining, gradient alcohol dehydration, xylene clearing, and neutral balsam sealing before being observed microscopically.

### Statistical analysis

All data were expressed as the mean ± standard deviation (SD). The statistical analysis was conducted using GraphPad Prism software (version 9.0). *p* < 0.05 was considered as statistical significance.Comparisons between the two groups were evaluated by one-way analysis of variance (ANOVA).

## Results

### XLW improved thyroid function in rats with hyperthyroidism

**Fig. 2:**
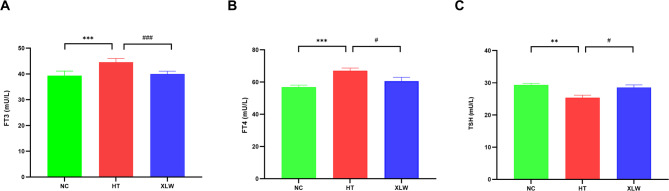
Serum FT3, FT4, and TSH levels of rats in each group (**A**) FT3 level, (**B**) FT4 level, and (**C**) TSH level. **p, ***p < 0.01 versus NC group; ^#^*p* < 0.05, ^###^*p* < 0.001 versus HT group; *n* = 6 or 8

The serum levels of FT3, FT4, and TSH in each group are illustrated in Fig. 2. In the HT group, FT3 (*p* < 0.001) and FT4 (*p* < 0.001) levels increased significantly when compared to the NC group. Additionally, TSH levels decreased significantly in the HT group compared to the NC group (*p* < 0.01). After XLW intervention, serum FT3 (*p* < 0.001) and FT4 (*p* < 0.05) levels decreased significantly, while TSH levels were significantly higher than those in the HT group (*p* < 0.05). These findings indicated that XLW could help restore the levels of FT3, FT4, and TSH to their normal levels.

**Fig. 3:**
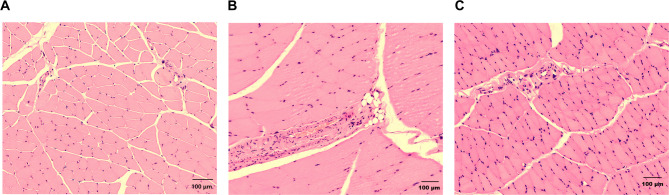
Morphological changes of the extraocular muscles in H&E staining. (**A**) NC group, ×20 (**B**) HT group, ×20, and (**C**) XLW group, ×20

### XLW alleviated the damage of extraocular muscles in rats with hyperthyroidism

HE staining can effectively reveal the morphological changes. As illustrated in Fig. 3, the extraocular muscles in normal rats demonstrated a strongly eosinophilic staining pattern with parallel arrangement of pathological structures. The connective tissue appeared clear, while the muscle fibers exhibited a band-like structure with a normal morphology and parallel arrangement. The cytoplasm displayed eosinophilia, along with multiple oval basophilic nuclei located in the periphery. Periodic transverse stripes were observed between light and dark bands. In the HT rat group, we observed infiltration of lymphocytes, monocytes, plasma cells, macrophages, adipose tissue, and fibroblasts proliferation in the extraocular muscle tissues, including the muscles themselves, the mesentery, and the sarcolemma. The eye muscle fibers were thickened, degenerated, destroyed, and fractured, resulting in a blurred texture and loss of muscular features. After treatment, we found that XLW resulted in improvements in inflammatory cell infiltration within the extraocular muscles, as well as adipose tissue infiltration and connective tissue hyperplasia within muscles and surrounding interstitium and sarcolemmal regions. Manifestations such as thickening, degeneration, destruction, and fractures of the ocular muscle fibers, along with the blurring and disappearance of textures, were also improved.

### Results of network pharmacology-based analysis

#### Assessment of active components and associated targets

Screening through the TCMSP identified 27 bio-active components from the three herbs of XLW: 9 from XS, 11 from XKC, and 7 from ZBM (Table 2). The gene names of potential targets for each active ingredient were standardized, resulting in 42 targets for XS, 183 for XKC, and 25 for ZBM. Furthermore, 18 candidate targets for ML were obtained from the TCMID database (Table 3).

**Table 2:** The candidate compounds of ZBM, XS, and XKC from TCMSP

HERB	Mol ID	Molecule Name	OB (%)	DL
ZBM	MOL001004	Pelargonidin	37.99	0.21
	MOL000358	Beta-sitosterol	36.91	0.75
	MOL004440	Peimisine	57.4	0.81
	MOL004443	Zhebeiresinol	58.72	0.19
	MOL004444	Ziebeimine	64.25	0.7
	MOL004446	Methoxyl-2-acetyl-3-methyl-1, 4-Naphthoquinone-8-O-beta-D-Glucopyranoside	33.31	0.57
	MOL004450	Chaksine	65.63	0.66
XS	MOL001925	paeoniflorin_qt	68.18	0.4
	MOL002222	Sugiol	36.11	0.28
	MOL000358	Beta-sitosterol	36.91	0.75
	MOL000359	Sitosterol	36.91	0.75
	MOL007657	scropolioside A_qt	38.63	0.77
	MOL007658	14-Deoxy-12(R)-sulfoandrographolide	62.57	0.42
	MOL007659	scropolioside D	36.62	0.4
	MOL007660	scropolioside D_qt	33.17	0.82
	MOL007662	harpagoside_qt	122.87	0.32
XKC	MOL000358	Beta-sitosterol	36.91	0.75
	MOL000422	Kaempferol	41.88	0.24
	MOL004355	Spinasterol	42.98	0.76
	MOL000449	Stigmasterol	43.83	0.76
	MOL004798	Delphinidin	40.63	0.28
	MOL000006	Luteolin	36.16	0.25
	MOL006767	Vulgaxanthin-I	56.14	0.26
	MOL006772	Poriferasterol monoglucoside_qt	43.83	0.76
	MOL006774	Stigmast-7-enol	37.42	0.75
	MOL000737	Morin	46.23	0.27
	MOL000098	Quercetin	46.43	0.28

**Table 3:** The relevant information of ML from TCMID

HERB	Target ID	Gene Symbol	Target Name	Target Class
ML	TCMT1	EHMT2	Histone-lysine N-methyltransferase, H3 lysine-9 specific 3	Transferase
	TCMT18	GAA	Lysosomal alpha-glucosidase	Hydrolase
	TCMT132	BCHE	Butyrylcholinesterase	Hydrolase
	TCMT131	ACHE	Acetylcholinesterase	Hydrolase
	TCMT125	GBA	Beta-glucocerebrosidase	Hydrolase
	TCMT101	PTPN1	Protein-tyrosine phosphatase 1B	Hydrolase
	TCMT907	FDPS	Farnesyl diphosphate synthase	Lyase
	TCMT158	EPHX2	Epoxide hydratase	Lyase
	TCMT17	POLB	DNA polymerase beta	Transferase
	TCMT2	MAPT	Microtubule-associated protein tau	Unclassified
	TCMT8	KDM4E	Lysine-specific demethylase 4D-like	Oxidoreductase
	TCMT34	CYP2D6	Cytochrome P450 2D6	Oxidoreductase
	TCMT33	CYP3A4	Cytochrome P450 3A4	Oxidoreductase
	TCMT223	AKR1B1	Aldose reductase	Oxidoreductase
	TCMT747	ABCG2	ATP-binding cassette sub-family G member 2	Transporter
	TCMT629	ABCC1	Multidrug resistance-associated protein 1	Transporter
	TCMT80	MAPK1	MAP kinase ERK2	Kinase
	TCMT82	ESR1	Estrogen receptor alpha	Nuclear receptor

### “Herb-active ingredient-core target” network construction

**Fig. 4:**
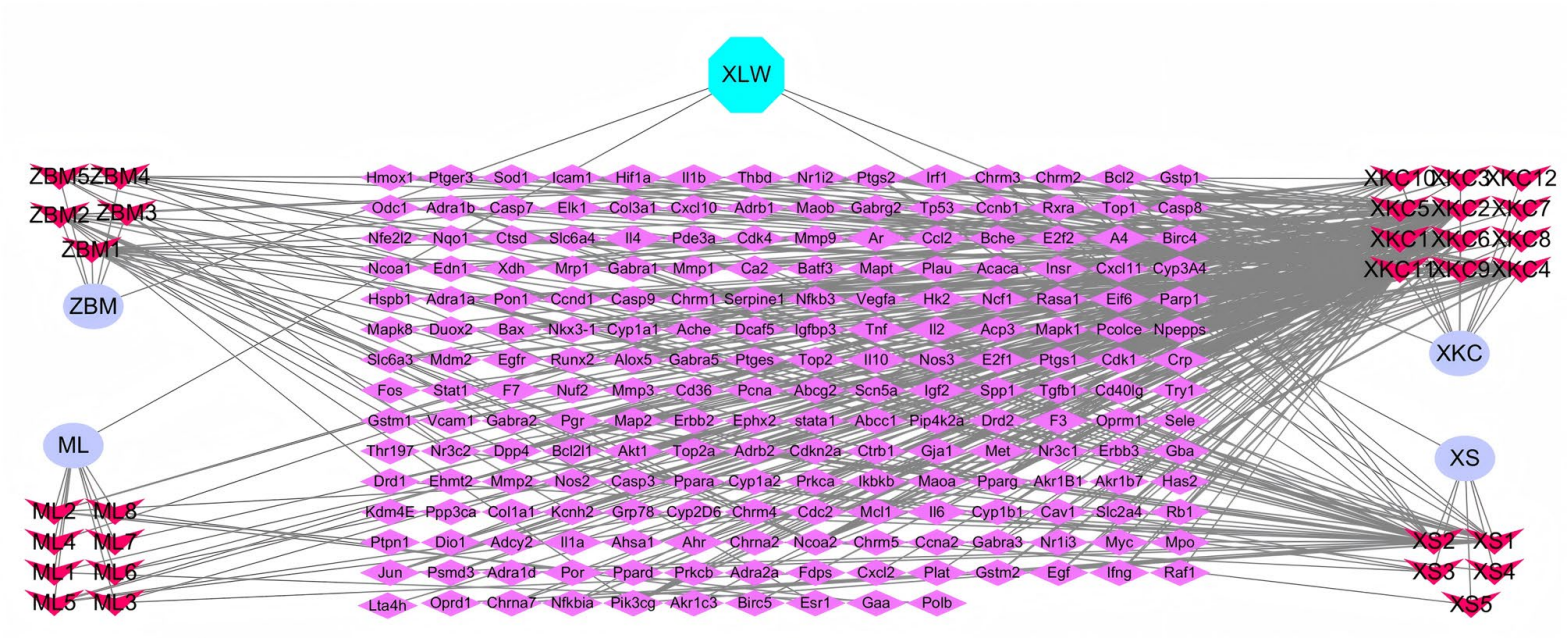
Herb-compound-target network

Figure 4 illustrates a “drug-active ingredient-core target” network diagram created by inputting XLW’s active ingredients and potential action targets into Cytoscape (version 3.9.1). The network comprised 480 edges and 241 nodes. Active components with the highest degree values were PTGS2 (14), Ncoa2 (13), Ptgs1 (11), and Pgr (10). This network illustrates XLW’s regulatory features, characterized by its multi-component, multi-target, and multi-pathway interactions, underscoring its distinct therapeutic mechanisms and benefits. Using Network Analyzer, the top four nodes by degree size were identified as follows: Quercetin (XKC11) with a degree of 132, kaempferol (XKC2) with 57, luteolin (XKC6) with 54, and sugiol (XS2) with 35. Key targets with a high degree of centrality can significantly influence the pharmacological effects of drugs in treating TAO.

### Screening of overlapping targets between drugs and diseases

**Fig. 5:**
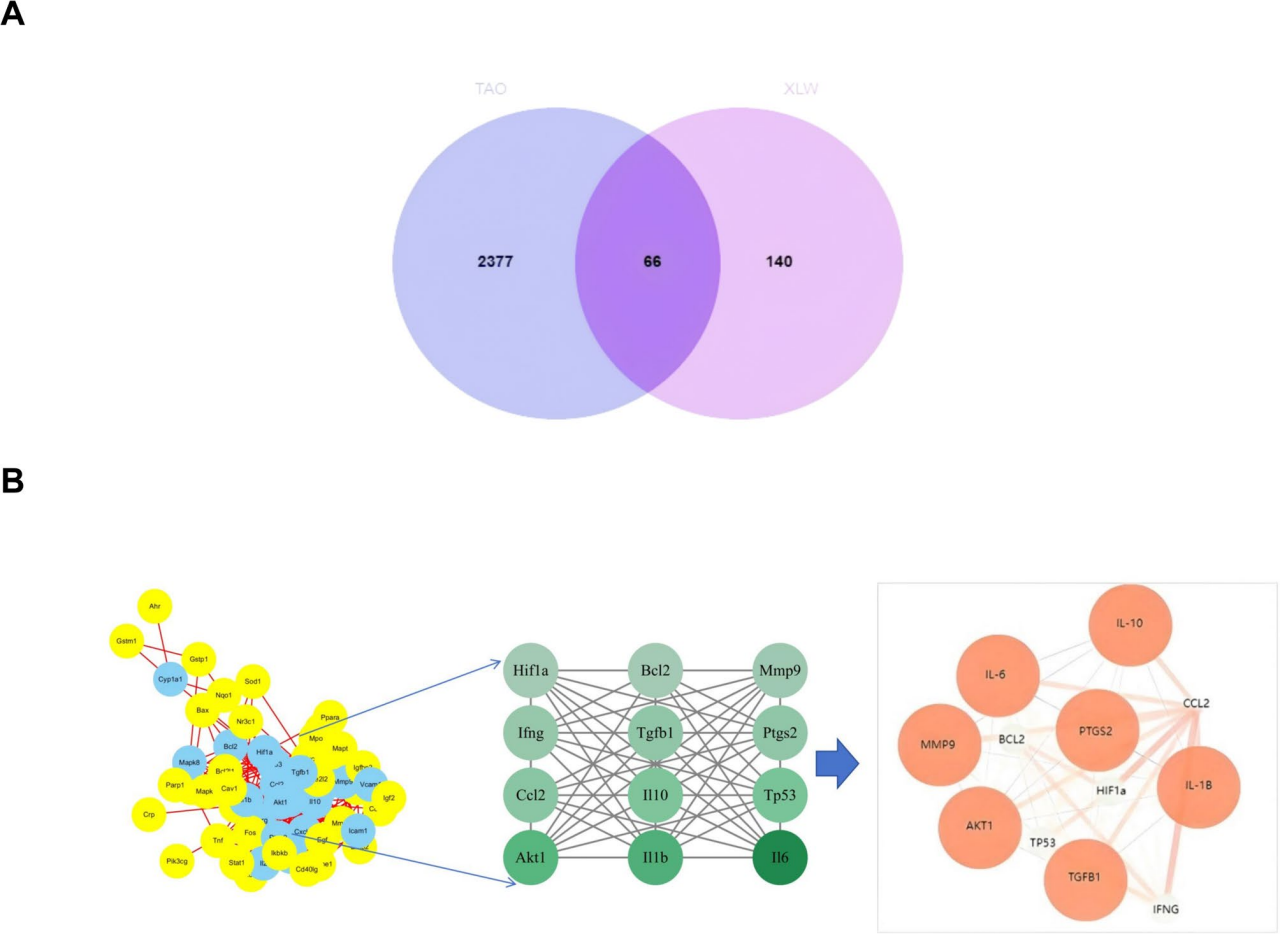
Network analysis diagram. (**A**) Venn diagram for the obtained targets of TAO and XLW. (**B**) PPI diagram of extraocular muscle lesions caused by XLW treatment of hyperthyroidism

Using Genecards, DisGeNET, OMIM, and DrugBank databases, we obtained 2443 targets of TAO. We imported 206 drug targets and 2,443 disease targets into an online platform (http://www.bioinformatics.com.cn/) for data analysis and visualization. Finally, a Venn diagram was drawn to identify 66 intersected targets between XLW and TAO (Fig. 5A).

### PPI network analysis

We utilized the Cytoscape Network Analyzer from the STRING database to examine the topological structure of the PPI network, focusing on the 66 overlapping targets that were imported into the STRING database for analysis. Subsequently, we performed module analyses on the entire network using Centisape (version 2.2) (Fig. 5B).

The network topology analysis of the target graph revealed that it consists of 65 nodes and 220 edges. There was a stronger relationship between the degree values and the sizes and shading of the nodes, indicating the greater importance of targets in this relationship. According to the scores of Betweenness, Closeness, and Degree unDir, 12 therapeutic nodes were identified as core therapeutic targets for TAO treatment (Table 4).

**Table 4:** Therapeutic targets screened from the PPI network

Name	Degree unDir	Betweenness unDir	Closeness unDir
AKT1	33	431.7716857	0.0125
PTGS2	24	59.00027641	0.011235955
BCL2	21	116.7683574	0.010989011
IL10	27	100.9923074	0.011627907
IL1b	34	259.8822202	0.012820513
CCL2	25	64.17895899	0.010869565
IFNG	23	56.33265574	0.010989011
IL6	41	522.3603024	0.014084507
MMP9	22	66.9961312	0.010989011
TGFB1	24	102.1551396	0.011235955
HIF1ɑ	21	145.6036806	0.010989011
TP53	28	475.1513339	0.012195122

### KEGG pathway enrichment and GO function analysis

**Fig. 6:**
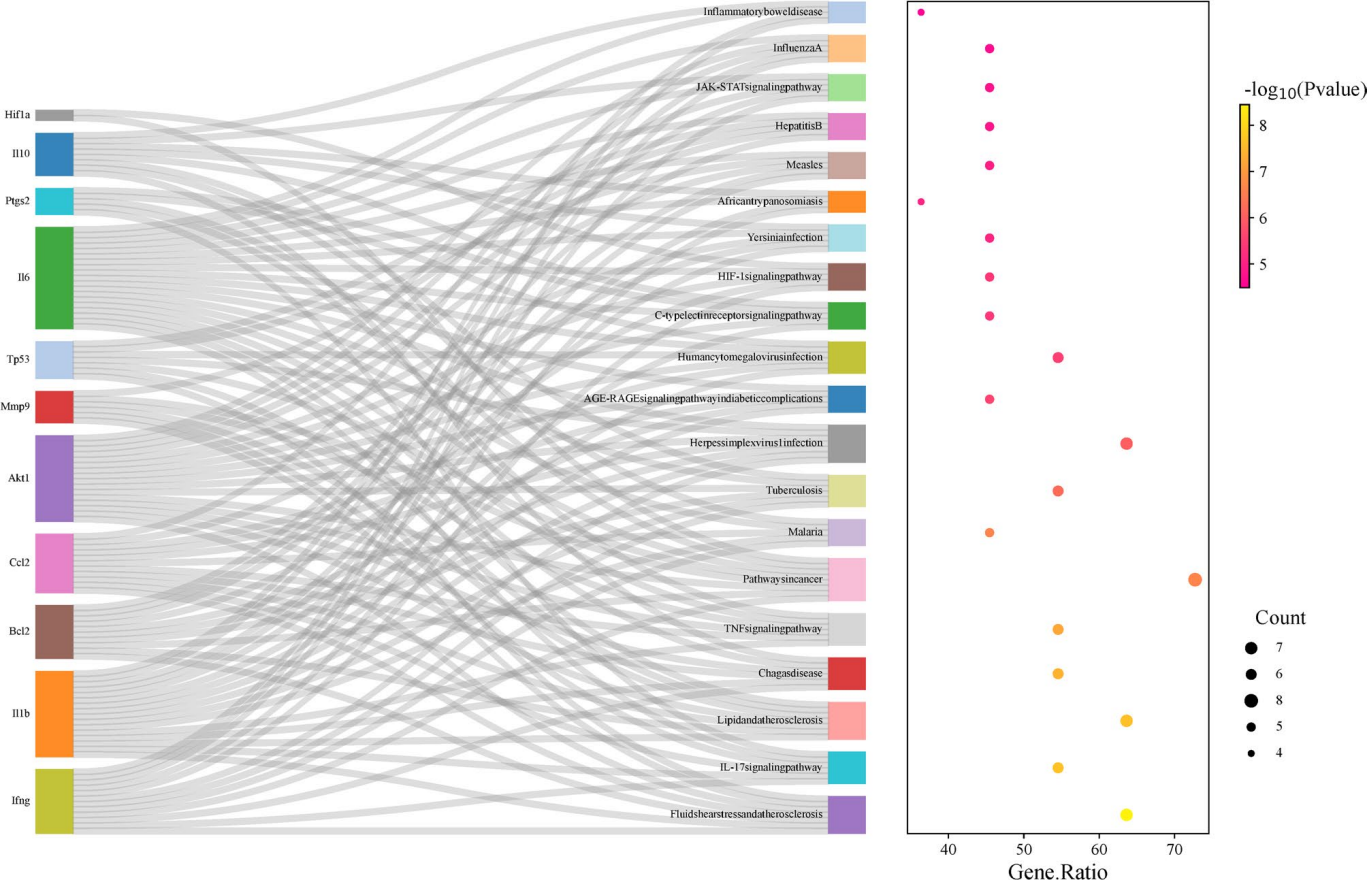
Top 20 of the KEGG bubble diagram. Bubble size represented the number of core targets involved in the pathway, and the color from red to yellow indicated that the P-value was from small to large

The potential therapeutic target genes were analyzed for GO enrichment and KEGG pathways to better understand the biological effects of the XLW treatment with TAO. Based on the p-value (*p* < 0.05), the top 20 KEGG pathways were displayed in a bubble chart (Fig. 6), which included HIF1, IL-17, and TNF signaling pathways.

**Fig. 7:**
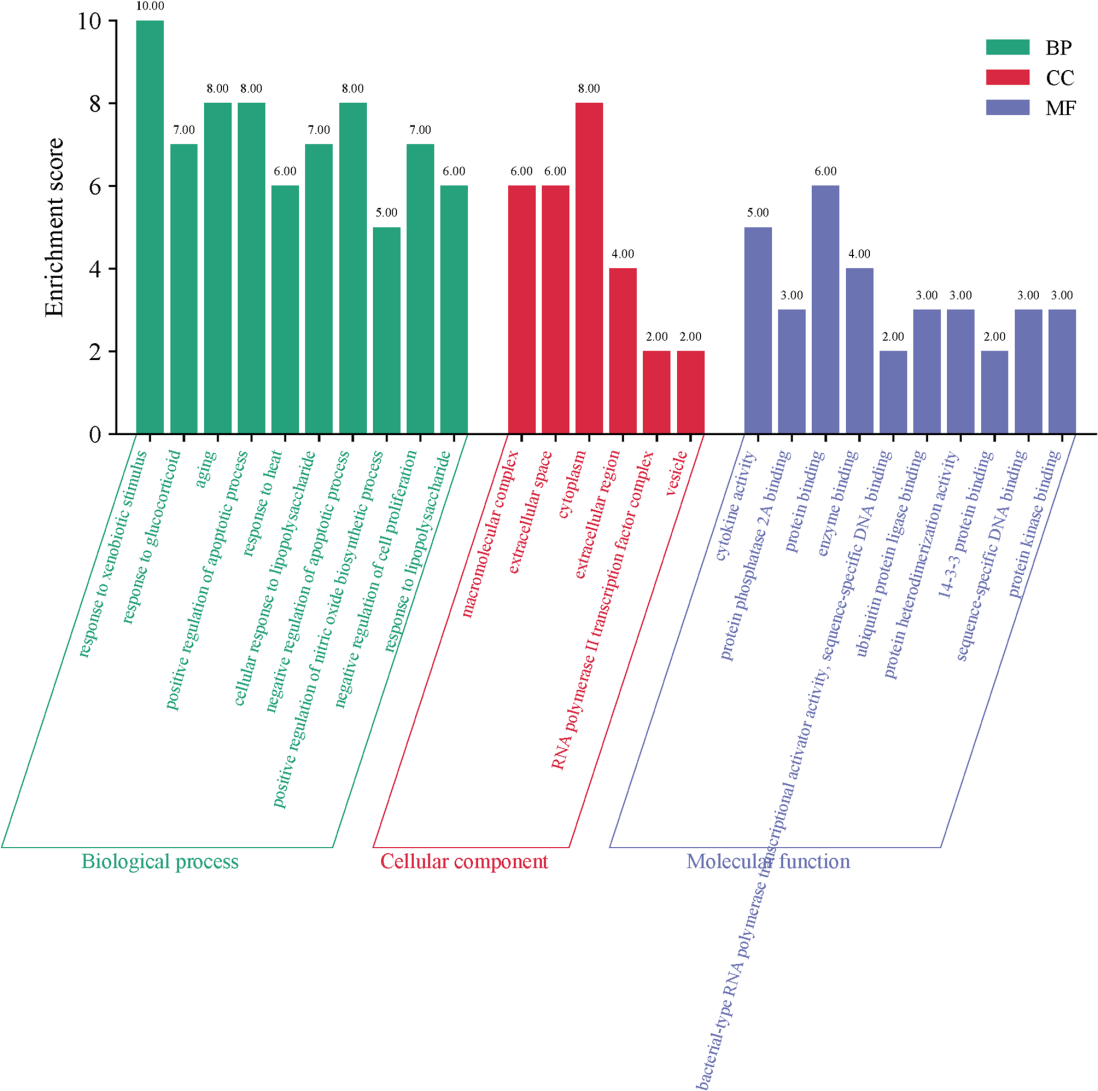
Top 10 terms for BP, CC, and ME

GO annotation is categorized into three terms: Biological process (BP), cellular component (CC), and molecular function (MF). This classification includes 216 BP, 18 MF, and 6 CC terms, with the top 10 items illustrated in Fig. 7. The primary BP identified include responses to xenobiotic stimuli, glucocorticoids, aging, positive regulation of apoptosis, responses to heat, and cellular responses to lipopolysaccharide. Central MF primarily included enriched cytokine activity, protein phosphatase 2 A binding, protein binding, enzyme binding, and bacterial-type RNA polymerase transcriptional activator activity. The main CC involved were macromolecular complex, extracellular space, cytoplasm, extracellular region, RNA polymerase II transcription factor complex, and vesicles.

### Network diagram of “formulae-herb − compound-target − disease-pathway”

**Fig. 8:**
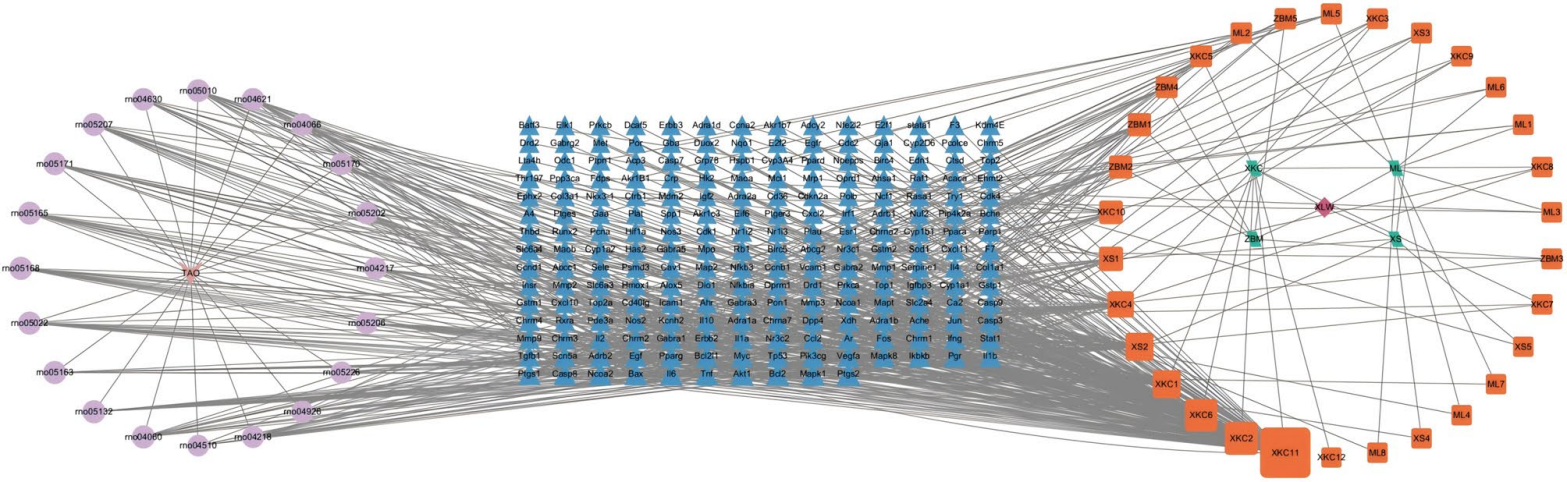
Formulae-herb-compound-target-pathway-disease

Figure 8 illustrates the “Formulae-Herb-Compound-Target-Pathway-Disease” network that was generated using Cytoscape (version 3.9.1). The diagram employs different shapes and colors to represent various elements. The pale pink diamond denotes XLW, while the green parallelogram indicates the herb names of XLW. The red square signifies the active ingredients of the herbs, the blue triangle represents the core target, and the violet circle illustrates the pathway. Lastly, the light pink V-shape depicts the disease name. The network comprised 242 nodes and 718 edges. A node with a higher degree value has a larger size, indicating greater importance in the network. It is concluded that XLW has the potential to alleviate TAO by regulating multiple components, targets, and signaling pathways.

### Experimental evaluations of mechanisms of XLW against extraocular muscle lesions caused by hyperthyroidism

#### Effect of XLW on OS in extraocular muscles of rats with hyperthyroidism

**Fig. 9:**
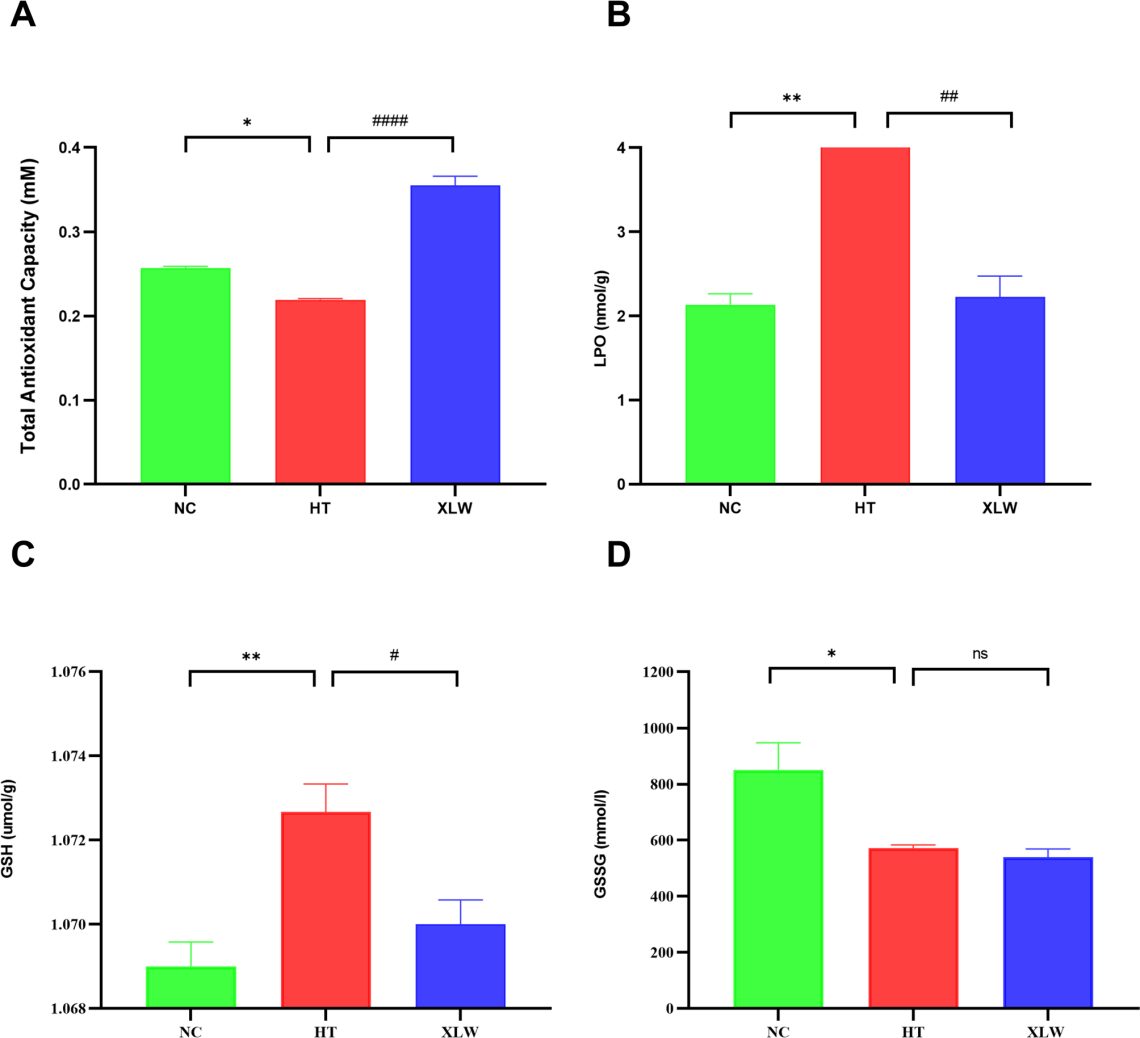
Effects of XLW on the levels of OS status in rat extraocular muscle tissues. (**A**) T-AOC, (**B**) LPO, (**C**) GSH, and (**D**) GSSG. *p < 0.05, **p < 0.01 versus NC group; ^#^*p* < 0.05, ^##^*p* < 0.001 versus HT group; ns: not significant; *n* = 6 or 8

The levels of redox markers, including T-AOC, LPO, GSH, and GSSG, were examined in the extraocular muscle tissues of rats from each group. Figure 9 illustrates that, in the HT group, T-AOC and GSSG levels were lower compared to the NC group (*p* < 0.05). Conversely, LPO (*p* < 0.01) and GSH (*p* < 0.05) levels increased significantly in the HT group. Following XLW intervention, T-AOC levels exhibited a notable rise (*p* < 0.01), while LPO and GSH expressions exhibited a significant reduction (*p* < 0.05). The findings suggested that XLW supplementation enhances the T-AOC of rats and concurrently restores LPO and GSH expressions to normal levels.

### Effect of XLW on HIF1α and PTGS2 expressions in rat extraocular muscles

**Fig. 10:**
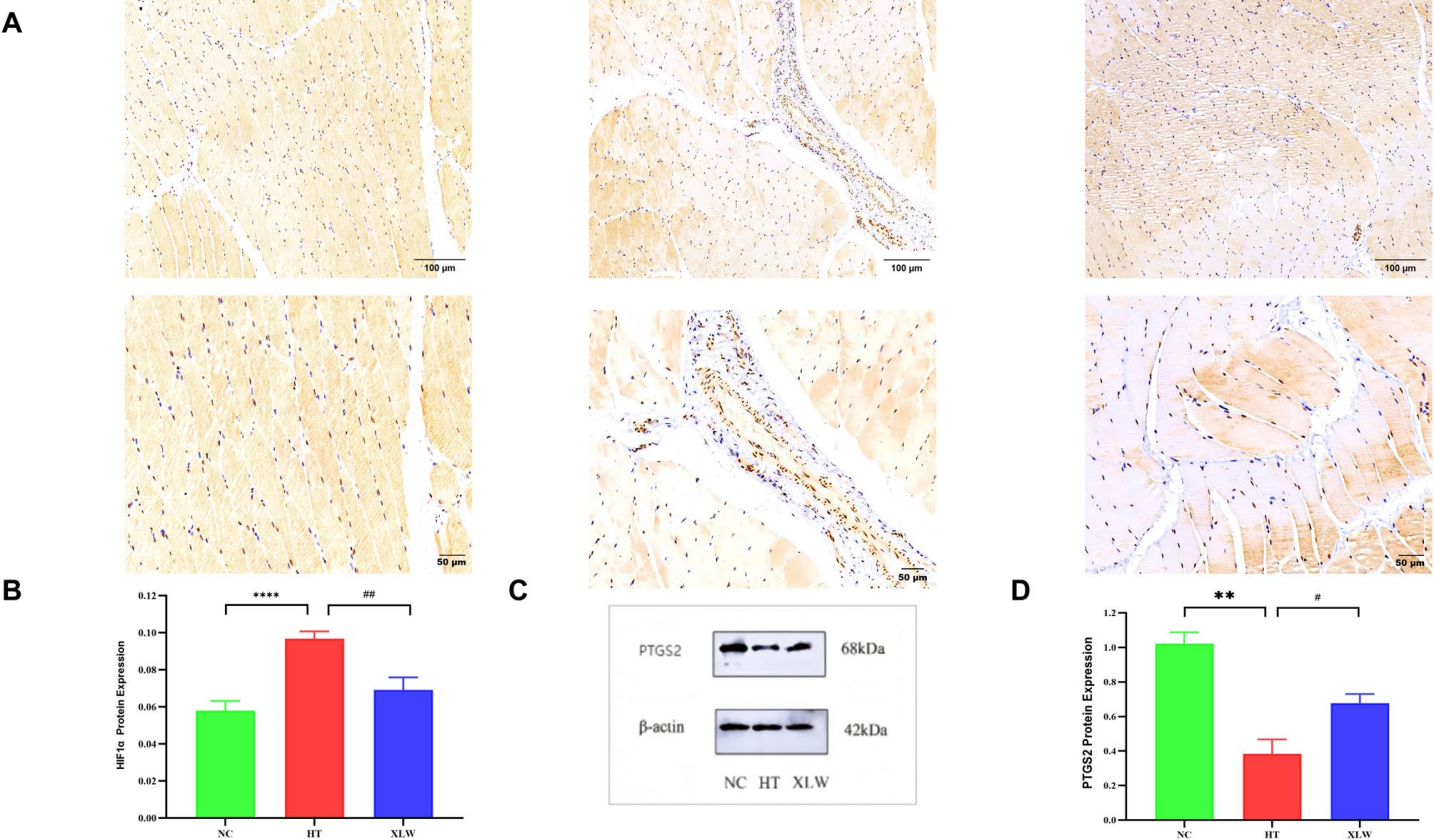
Effects of XLW on HIF1α and PTGS2 expressions in rat extraocular muscle tissues. (**A**) The immunohistochemical slice of HIF1α, (**B**) HIF1α immunohistochemical quantitative analysis, (**C**) WB of PTGS2 levels, and (**D**) quantification of the WB in PTGS2. ^**^p < 0.01, ^****^p < 0.0001 versus NC group; ^#^*p* < 0.05, ^##^*p* < 0.01 versus HT group; *n* = 6 or 8

Network analysis identified HIF1α and PTGS2 as core targets, with PTGS2 exhibiting the highest degree value. KEGG enrichment analysis suggested the involvement of the HIF1 signaling pathway, which is closely associated with HIF1α. Consequently, we observed the presence of HIF1ɑ and PTGS2 in extraocular muscles. As illustrated in Fig. 10, the immunohistochemistry results revealed that the expression of HIF1ɑ in the HT group was significantly higher in comparison to the NC group (*p* < 0.0001). Furthermore, there was a notable increase in HIF1α expression following the XLW intervention (*p* < 0.01). Similarly, WB analysis demonstrated that XLW significantly enhanced the expression level of PTGS2 protein (*p* < 0.05). Overall, these findings suggest that XLW may improve the condition of extraocular muscles in TAO-rats by modulating HIF1α and PTGS2 expressions.

### Effect of XLW on NOX2, NOX4, and GPX4 expressions in rat extraocular muscles

**Fig. 11:**
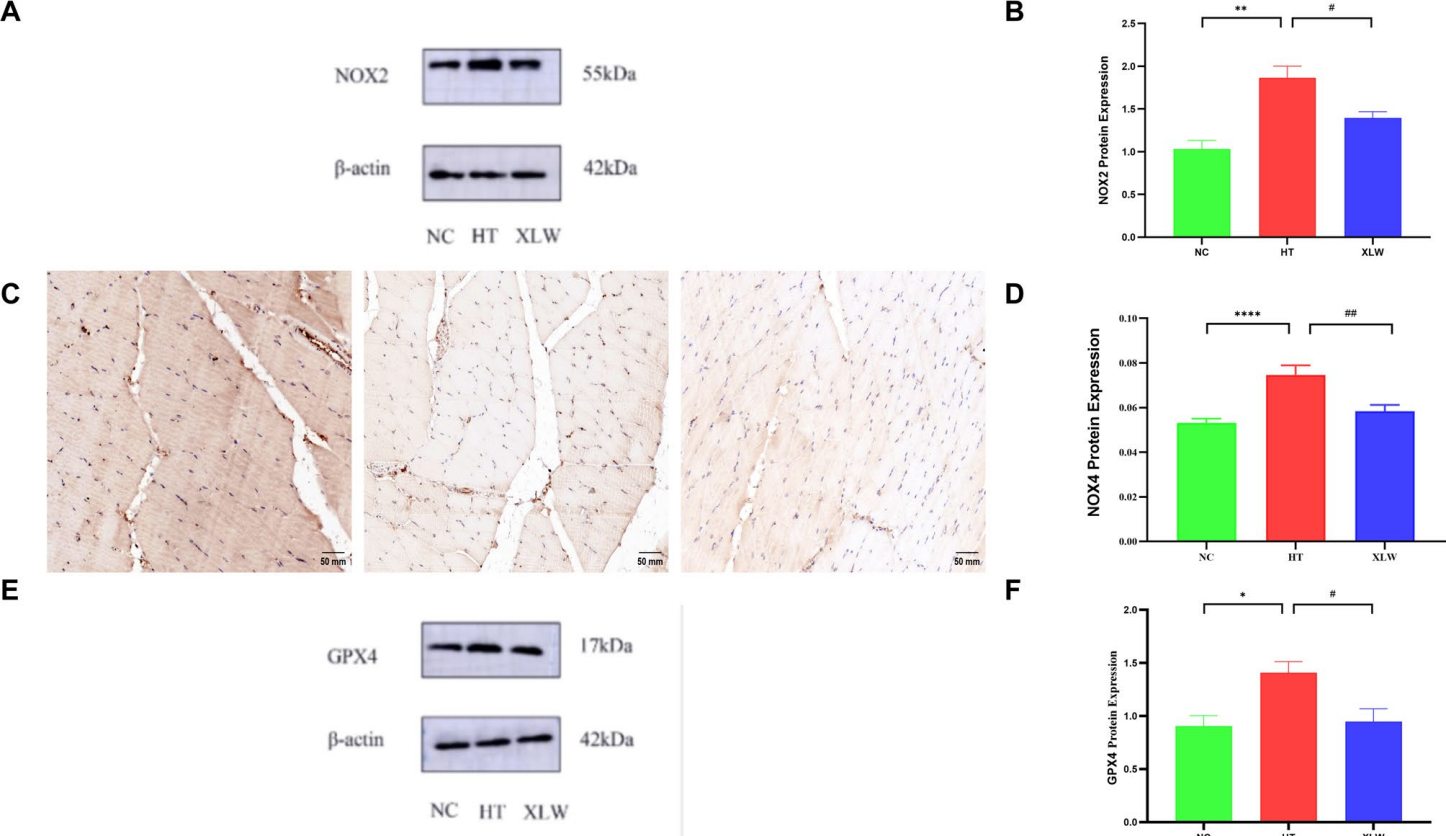
Effects of XLW on the expression of NOX2, NOX4, and GPX4 in rat extraocular muscle. (**A**) WB of NOX2 levels, (**B**) quantification of the WB in NOX2, (**C**) the immunohistochemical slice of NOX4, (**D**) NOX4 immunohistochemical quantitative analysis, (**E**) WB of GPX4 levels, and (**F**) quantification of the WB in GPX4. ^*^p < 0.05, ^**^p < 0.01, ^****^p < 0.0001 versus NC group; ^#^*p* < 0.05, ^##^*p* < 0.01 versus HT group; *n* = 6 or 8

Given that OS may be a mechanism through which XLW treats TAO, we acknowledged NOX2 and NOX4 as major sources of ROS besides mitochondria. As GPX4 plays a crucial role in antioxidant defense and serves as an indicator of antioxidant status [24, 25]we investigated the expression levels of NOX2, NOX4, and GPX4. As depicted in Fig. 11, the HT group exhibited a significant increase in protein levels of NOX2 (*p* < 0.01), NOX4 (*p* < 0.0001), and GPX4 (*p* < 0.05) in the extraocular muscles compared to the NC group. Protein expression levels decreased after XLW treatment (*p* < 0.05), indicating that XLW may reduce NOX2, NOX4, and GPX4 expressions.

## Discussion

The chronic nature, prolonged duration, and tendency to recur make the pathogenesis of TAO difficult to understand, leading to a lack of specific medications for comprehensive treatment. Therefore, exploring new treatment strategies is crucial for the clinical management of TAO. TCM may serve as a useful resource for TAO therapies. According to TCM theory, XLW has the effects of Ruanjian Sanjie and Ziyin Jianghuo. This study combines network analysis and animal experiments to comprehensively analyze the protective effects and potential mechanisms of XLW against TAO, providing a basis for further application.

In this animal research, we found that intraperitoneal administration of L-thyroxine resulted in increased levels of serum FT3 and FT4, decreased TSH levels, and excessive OS in rats. This ultimately led to the development of extraocular myopathy. XLW demonstrated efficacy in improving serum thyroid hormone levels in hyperthyroid rats through its antioxidant properties.

OS occurs when ROS outpaces antioxidants, marked by high levels of ROS, low defense, and the presence of ocular by-products [9, 26]. In our study, we found that hyperthyroid rats exhibited a significant imbalance between pro- and antioxidant systems following intervention with L-thyroxine. This imbalance was demonstrated by a reduction in T-AOC and GSSG, accompanied by increased levels of GSH, GPX4, and LPO in extraocular muscle tissue. GSH exerts protective effects against OS [27]while GPX4 plays a crucial role in mitigating lipid peroxides during OS [28]. Additionally, the synthesis of GSH directly enhances GPX4 activity [29]. The GSH/GSSG redox balance reaction is extensively used as a biomarker for assessing OS [30]. The coordinated changes in LPO, GSH/GSSG, and GPX4 provide comprehensive evidence of XLW’s amelioration of oxidative injury. Although MDA/SOD were not assayed, LPO serves as a direct precursor to MDA, and T-AOC/GSH dynamics reflect SOD-related antioxidant capacity. These findings indicated that the extraocular muscle tissue of rats experienced severe OS, leading to a depletion of accumulated T-AOC and GSSG, while GSH and GPX4 levels were abnormally elevated. Moreover, XLW could help attenuate the OS response in hyperthyroid rats. This positive effect may be associated with the presence of quercetin [31]luteolin [32]kaempferide [33]rosmarinic acid [34]and β-sitosterol [35] found in XLW, indicating that natural medicines may play a role in reducing oxidative stress responses in hyperthyroid rats.

HIF1 functions as a master regulator of oxygen homeostasis. Under hypoxia, HIF1α accumulates and binds to HIF1β, forming an active HIF1 complex. This complex enhances orbital fat vascularization and adipocyte differentiation in fibroblasts [36, 37]. Hypoxia-induced OS triggers extraocular muscle self-protection, associated with HIF1α stabilization and activation [38]. Our findings revealed that the increase in HIF1 levels was positively correlated with the degree of TAO inflammatory activity. Hypoxia promotes the formation of blood vessels and adipocytes via HIF1, exacerbating TAO, which aligns with prior research [39]. However, after the intervention of TCM, the oxygen content increased significantly, accompanied by a decrease in protein expression. These observations suggest that XLW alters OS, benefiting TAO. In addition, bio-active ingredients such as luteolin can inhibit ROS production and deplete HIF 1α and PTGS2 activity [40]consistent with our experimental observation.

As reported, NOX2 and NOX4—downstream targets of HIF1α—are crucial for ROS production in TAO [41–44]. Our study revealed that increased levels of NOX2 and NOX4 in extraocular muscle tissue could trigger the development of TAO by activating OS products (as evidenced by LPO levels). This activation may be linked to the HIF1 signaling pathway. NOX2 and NOX4 primarily produce oxygen free radicals. The continuous production of these oxygen-free radicals in the extraocular muscle tissue of hyperthyroid rats leads to a decrease in oxygen content and OS damage. This process produces numerous substrates, with heightened NOX activation inducing damage in extraocular muscle tissue of hyperthyroid rats via lipid per-oxidation [45]. Besides, consistent with previous findings, quercitrin reduces the expression of NOX2 and NOX4 [46]. In this study, NOX2 and NOX4 expressions were found to be significantly reduced after XLW therapy. This provides strong evidence supporting XLW’s effectiveness in alleviating symptoms in rats with TAO.

However, this study has several limitations. First, only the effective components of traditional Chinese medicine were obtained by using public databases, and high-performance liquid chromatography was not used to clarify the components. While database screening efficiently prioritizes mechanistically relevant targets, future studies will integrate HPLC-MS to quantify specific compounds in XLW extracts and correlate their levels with in vivo efficacy. Second, the study revealed no clear dose-response relationship between the drug and the disease. Third, other signaling pathways and key genes may also significantly contribute to the effects of XLW on hyperthyroidism-induced extraocular muscle diseases. Our future research will focus on developing a rapid, sensitive, and efficient methodology to address these limitations. This will help us generate new insights for the effective and rational use of ancient classical prescriptions, as well as for the research and development of new pharmaceuticals.

## Conclusion

According to network pharmacological analysis, the effective mechanisms of XLW in extraocular muscle lesions caused by hyperthyroidism involve the regulation of targets in various BPs, particularly OS. The antioxidant effects of XLW in the L-thyroxine-induced hyperthyroid rat model appear to be mediated through the regulation of specific targets, including HIF1α, NOX4, NOX2, PTGS2, and GPX4. This research suggests that XLW could serve as an effective therapeutic agent for extraocular muscle lesions caused by hyperthyroidism. These findings represent a promising advancement in understanding the impact of herbal formulas on TAO.

## Electronic supplementary material

Below is the link to the electronic supplementary material.

Supplementary Material 1

## Data Availability

No datasets were generated or analysed during the current study.
